# Randomized controlled trials: who fails run-in?

**DOI:** 10.1186/1745-6215-16-S2-O78

**Published:** 2015-11-16

**Authors:** Judy R Rees, Leila A Mott, Elizabeth L Barry, John A Baron, Janet L Peacock

**Affiliations:** 1 Geisel School of Medicine at Dartmouth, Department of Epidemiology, Hanover, NH, USA; 2 Division of Health and Social Care Research, King’s College, London, UK; 3 NIHR Biomedical Research Centre at Guy's and St Thomas' NHS Foundation Trust and King's College, London, UK; 4 University of North Carolina at Chapel Hill, NC, USA

## Background

Early identification of enrollees at risk of poor adherence and run-in failure (RIF) may present opportunities to increase trial efficiency and generalizability.

## Methods

We conducted a factorial-design randomized, controlled trial of calcium and vitamin D to prevent colorectal adenoma recurrence. At the enrolment interview, study coordinators at 11 centers collected demographic and medical information and participants’ beliefs about the study tablets. Participants also completed two self-administered questionnaires (SAQ) before a three-month single-blinded placebo run-in. Eligible participants were then randomized to calcium, vitamin D, both or neither; women electing to take calcium were randomized to vitamin D or placebo. *A priori*, we considered three subgroups: men (N=1606) and women (N=301) in the full factorial randomization and women in the 2-arm randomization (N=666).

## Results

Overall, 314 of 2,573 (12%) enrollees potentially eligible for randomization failed run-in due to poor adherence (took <80% tablets) or refusal to participate. In multivariable models in the largest subgroup (males), RIF was associated with younger age (adjusted odds ratio per 5 years 0.85; 95% CI 0.76-0.96), single marital status (1.67; 1.12-2.49), any missing data on the SAQs (2.05; 1.46-2.86) study center (p<0.0001) and perceived toxicity report (12.86; 5.41-30.56). Across all three subgroups, the latter three factors were most consistently associated with RIF but other factors are described which vary by subgroup.

## Conclusions

The most consistent predictors of RIF were perceived toxicities, missing data on self-administered questionnaires, and study center. The latter two findings relate to study coordinator oversight, and present potential opportunities to improve adherence during run-in.

